# Supplementary material for: Epigenetic drift underlies epigenetic clock signals, but displays distinct responses to lifespan interventions, development, and cellular dedifferentiation
Source: Aging (Albany NY). 2024 Jan 26;16(2):1002–20. doi: 10.18632/aging.205503 (PMC10866415; doi:10.18632/aging.205503)
Supplement: Supplementary Figures [file aging-16-205503-s001.pdf]

SUPPLEMENTARY FIGURES

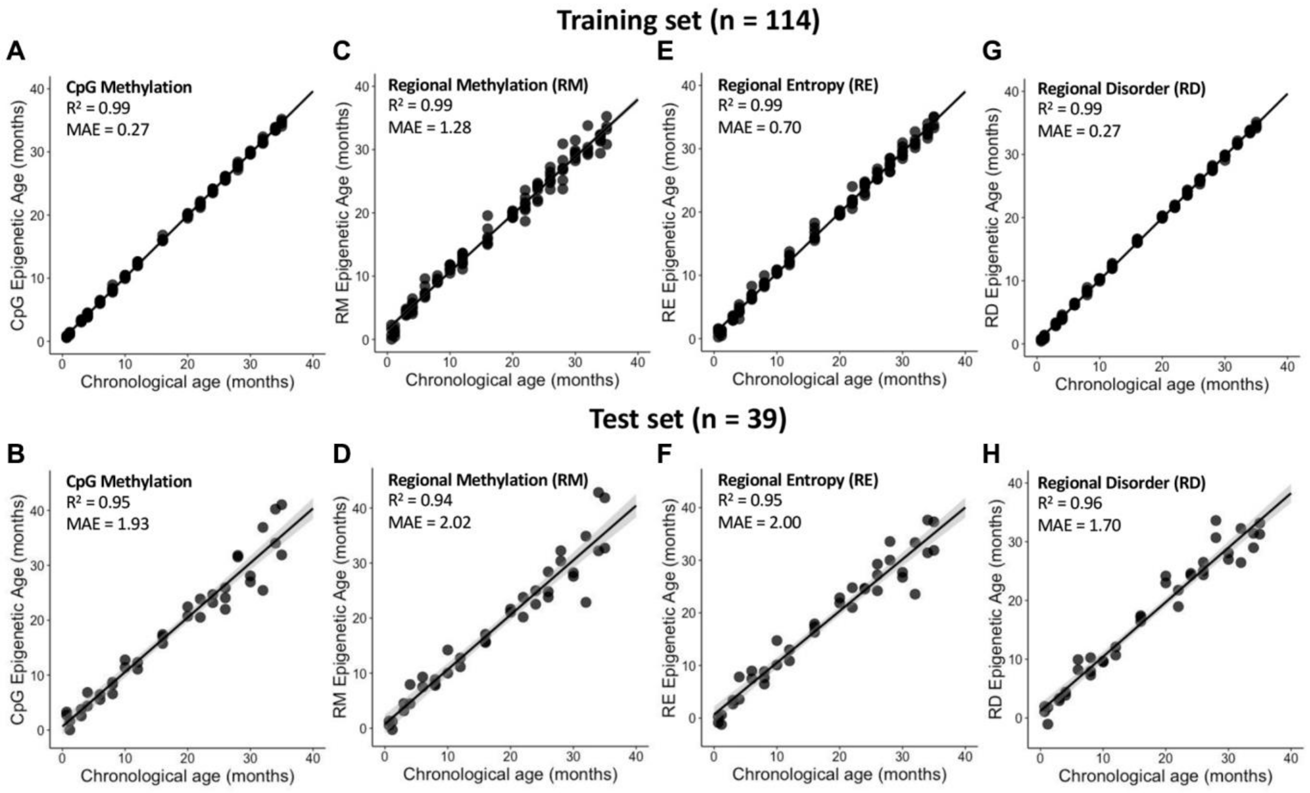

I

Clock type	CpG	RM	RE	RD
Predictors	129 CpGs	59 regions	86 regions	121 regions

Supplementary Figure 1. Representative epigenetic clocks from each datatype. (**A**, **B**) show results for the epigenetic clock constructed using CpG methylation data, (**C**, **D**) show results for the epigenetic clock constructed using regional methylation (RM) data, (**E**, **F**) show results from an epigenetic clock based on regional entropy (RE) data, and (**G**, **H**) show results from the epigenetic clock based on regional disorder (RD) data. Panel (**I**) indicates the number of predictors comprising each clock.

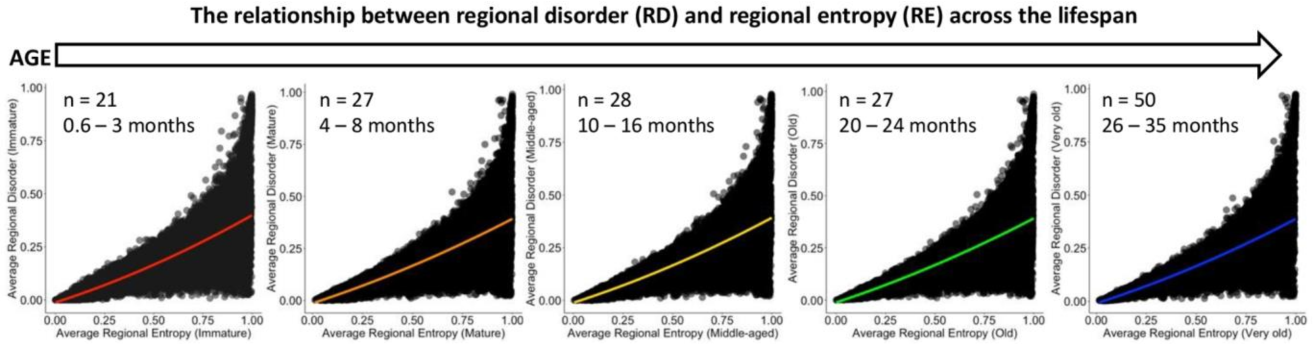

Supplementary Figure 2. The relationship between average regional disorder and average regional entropy across the lifespan of mice. Samples are broken up into distinct life stages which are denoted by different colors (immature – red, mature – orange, middle-aged – yellow, old – green, and very old – blue).

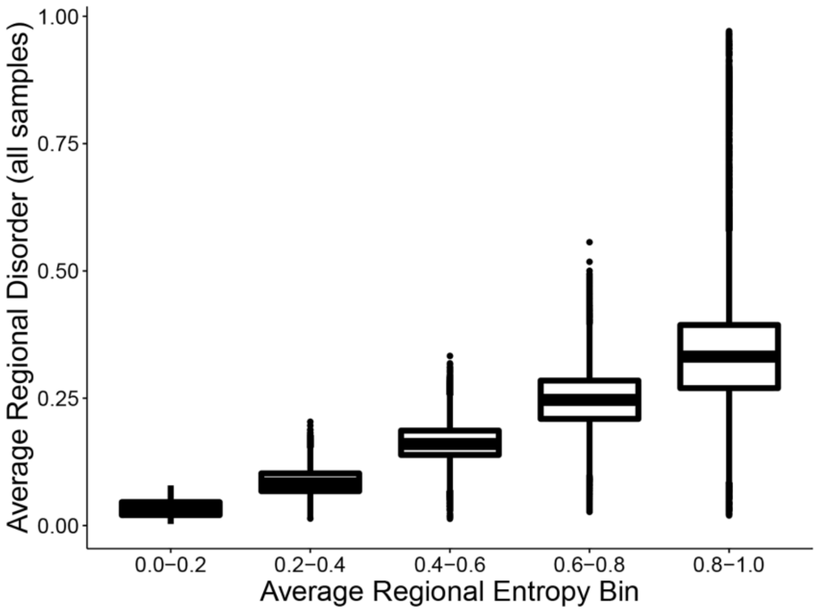

Supplementary Figure 3. Variation in average regional disorder increases with increasing values of average regional entropy.
