# Supplementary material for: Epigenetic drift underlies epigenetic clock signals, but displays distinct responses to lifespan interventions, development, and cellular dedifferentiation
Source: Aging (Albany NY). 2024 Jan 26;16(2):1002–20. doi: 10.18632/aging.205503 (PMC10866415; doi:10.18632/aging.205503)
Supplement: Supplementary Tables [file aging-16-205503-s002.pdf]

SUPPLEMENTARY TABLES

Supplementary Table 1. Gene ontology results from regions with age-associated gains in disorder.

Source	GO term name	GO ID	Adjusted *p*-value
GO:MF	DNA-binding transcription factor activity, RNA polymerase II-specific	GO:0000981	1.76E-28
GO:MF	DNA-binding transcription factor activity	GO:0003700	7.58E-28
GO:MF	sequence-specific double-stranded DNA binding	GO:1990837	5.72E-23
GO:MF	double-stranded DNA binding	GO:0003690	4.64E-21
GO:MF	sequence-specific DNA binding	GO:0043565	1.37E-20
GO:MF	RNA polymerase II transcription regulatory region sequence-specific DNA binding	GO:0000977	1.79E-20
GO:MF	transcription regulator activity	GO:0140110	5.41E-20
GO:MF	transcription cis-regulatory region binding	GO:0000976	7.85E-19
GO:MF	transcription regulatory region nucleic acid binding	GO:0001067	1.17E-18
GO:MF	RNA polymerase II cis-regulatory region sequence-specific DNA binding	GO:0000978	1.70E-17
GO:MF	cis-regulatory region sequence-specific DNA binding	GO:0000987	2.57E-17
GO:MF	binding	GO:0005488	8.47E-17
GO:MF	protein binding	GO:0005515	1.12E-15
GO:MF	DNA-binding transcription activator activity	GO:0001216	1.01E-11
GO:MF	DNA-binding transcription activator activity, RNA polymerase II-specific	GO:0001228	1.31E-11
GO:MF	gated channel activity	GO:0022836	1.80E-11
GO:MF	DNA binding	GO:0003677	8.54E-09
GO:MF	ion channel activity	GO:0005216	8.80E-09
GO:MF	voltage-gated cation channel activity	GO:0022843	1.21E-08
GO:MF	channel activity	GO:0015267	2.18E-08
GO:BP	nervous system development	GO:0007399	9.36E-84
GO:BP	neurogenesis	GO:0022008	1.71E-68
GO:BP	system development	GO:0048731	4.56E-67
GO:BP	multicellular organism development	GO:0007275	3.14E-65
GO:BP	generation of neurons	GO:0048699	4.99E-63
GO:BP	anatomical structure development	GO:0048856	4.01E-61
GO:BP	neuron differentiation	GO:0030182	3.05E-60
GO:BP	multicellular organismal process	GO:0032501	5.25E-58
GO:BP	developmental process	GO:0032502	5.17E-57
GO:BP	anatomical structure morphogenesis	GO:0009653	1.15E-54
GO:BP	cell-cell signaling	GO:0007267	1.09E-50
GO:BP	central nervous system development	GO:0007417	4.10E-48
GO:BP	neuron development	GO:0048666	2.56E-47
GO:BP	cell differentiation	GO:0030154	5.75E-45
GO:BP	cell development	GO:0048468	9.25E-45
GO:BP	cellular developmental process	GO:0048869	1.71E-44
GO:BP	neuron projection development	GO:0031175	2.60E-41
GO:BP	animal organ development	GO:0048513	3.67E-41
GO:BP	brain development	GO:0007420	2.06E-40
GO:BP	head development	GO:0060322	4.07E-40

Top 20 terms from molecular function (MF) and biological process (BP) based on significance values are shown.

Supplementary Table 2. Gene ontology results from regions with age-associated losses in disorder.

Source	GO term name	GO ID	Adjusted *p*-value
GO:MF	protein binding	GO:0005515	0.00063605
GO:MF	binding	GO:0005488	0.00159718
GO:MF	protein kinase activity	GO:0004672	0.00357349
GO:MF	phosphotransferase activity, alcohol group as acceptor	GO:0016773	0.01376325
GO:MF	transferase activity	GO:0016740	0.04052106
GO:BP	developmental process	GO:0032502	0.00019855
GO:BP	anatomical structure development	GO:0048856	0.00021537
GO:BP	multicellular organismal process	GO:0032501	0.00032362
GO:BP	anatomical structure morphogenesis	GO:0009653	0.00042435
GO:BP	response to stimulus	GO:0050896	0.00074356
GO:BP	signal transduction	GO:0007165	0.0017411
GO:BP	regulation of cellular process	GO:0050794	0.0030705
GO:BP	cellular response to stimulus	GO:0051716	0.0044849
GO:BP	signaling	GO:0023052	0.00807516
GO:BP	cell communication	GO:0007154	0.01493627
GO:BP	regulation of biological process	GO:0050789	0.01644778
GO:BP	biological regulation	GO:0065007	0.02094827
GO:BP	intracellular signal transduction	GO:0035556	0.03178697
GO:BP	multicellular organism development	GO:0007275	0.04073998
GO:MF	protein binding	GO:0005515	0.00063605
GO:MF	binding	GO:0005488	0.00159718
GO:MF	protein kinase activity	GO:0004672	0.00357349
GO:MF	phosphotransferase activity, alcohol group as acceptor	GO:0016773	0.01376325
GO:MF	transferase activity	GO:0016740	0.04052106
GO:BP	developmental process	GO:0032502	0.00019855
GO:BP	anatomical structure development	GO:0048856	0.00021537
GO:BP	multicellular organismal process	GO:0032501	0.00032362
GO:BP	anatomical structure morphogenesis	GO:0009653	0.00042435
GO:BP	response to stimulus	GO:0050896	0.00074356
GO:BP	signal transduction	GO:0007165	0.0017411
GO:BP	regulation of cellular process	GO:0050794	0.0030705
GO:BP	cellular response to stimulus	GO:0051716	0.0044849
GO:BP	signaling	GO:0023052	0.00807516
GO:BP	cell communication	GO:0007154	0.01493627
GO:BP	regulation of biological process	GO:0050789	0.01644778
GO:BP	biological regulation	GO:0065007	0.02094827
GO:BP	intracellular signal transduction	GO:0035556	0.03178697
GO:BP	multicellular organism development	GO:0007275	0.04073998

Terms from molecular function (MF) and biological process (BP) are shown.
